# Incidence of Epstein-Barr virus reactivation is elevated in COVID-19 patients

**DOI:** 10.1016/j.virusres.2023.199157

**Published:** 2023-06-26

**Authors:** Keishanne Danielle E. Bernal, Christopher B. Whitehurst

**Affiliations:** a Department of Pathology, Microbiology, and Immunology, New York Medical College, Basic Medical Sciences Building, 15 Dana Rd. Valhalla, NY 10595; b Westlake High School, 825 Westlake Dr., Thornwood, NY 10594

**Keywords:** COVID-19, Epstein-Barr virus, EBV, Reactivation, Coronavirus, SARS-CoV-2

## Abstract

COVID-19, an infectious respiratory illness, is caused by infection with the SARS-CoV-2 virus. Individuals with underlying medical conditions are at increased risk of developing serious illnesses such as long COVID. Recent studies have observed Epstein-Barr virus (EBV) reactivation in patients with severe illness or long COVID, which may contribute to associated symptoms. We determined the frequency of EBV reactivation in COVID-19 positive patients compared to COVID-19 negative patients. 106 blood plasma samples were collected from COVID-19 positive and negative patients and EBV reactivation was determined by detection of EBV DNA and antibodies against EBV lytic genes in individuals with previous EBV infection. 27.1% (13/48) of EBV reactivations, based on qPCR detection of EBV genomes, are from the COVID positive group while only 12.5% (6/48) of reactivations belonged to the negative group. 20/52 (42.30%) of the COVID PCR negative group had detectable antibodies against SARS-CoV-2 nucleoprotein (Np); indicative of past infection. A significantly higher SARS-CoV-2 Np protein level was found in the COVID-19 positive group. In conclusion, COVID-19 patients experienced increased reactivation of EBV in comparison to COVID negative patients.

## Highlights

• COVID-19 patients have increased incidence of Epstein-Barr Virus reactivation.
• Detection of EBV DNA is greater among COVID-19 positive patients (27.1% vs 12.5%).
• No statistical difference in CRP levels of COVID-19 positive vs. negative patients.
• No statistical difference in amount of EBV genomes in reactivated patient groups.

## 1 Introduction

COVID-19, which emerged in December of 2019, became a worldwide pandemic and has claimed over 6.6 million lives (World Health Organization 2023). While many infected individuals experience mild or moderate symptoms and recover in 7-10 days it has been reported that almost 16% of patients developed severe disease in a study conducted through January 29, 2020 (Guan et al., 2020). The mortality rate among severe cases of COVID has been reported to be as high as 61.5% (Yang et al., 2020). Since the initial emergence of COVID-19, four prominent variants have emerged: Alpha, Beta, Delta, and Omicron (Callaway, 2021). Omicron, the most recent and highly mutated variant of concern, was first identified in November 2021 and quickly became the dominant strain worldwide (Tian et al., 2022).

Reactivation of Epstein-Barr virus (EBV) has been reported among the critically ill and patients suffering from long COVID and EBV viremia has been correlated with COVID severity (Naendrup et al., 2022, Paolucci et al., 2021, Saade et al., 2021, Simonnet et al., 2021, Gold et al., 2021, Zubchenko et al., 2022, Vojdani et al., 2023). A longitudinal multi-omic study suggested that four main risk factors for developing long COVID are type-2 diabetes, SARS-CoV-2 RNAemia, specific auto-antibodies, and Epstein-Barr virus viremia (Su et al., 2022). Reactivation of EBV may contribute to COVID symptoms, severity, and length of illness. The mechanism by which EBV reactivation may contribute to COVID is not quite clear, however Verma et al reported that EBV lytic replication promotes ACE2 expression and therefore could facilitate entry of SARS-CoV-2 (Verma et al., 2021).

Epstein-Barr virus is one of nine known human herpesviruses and infects more than 90% of the world's population (Tzellos and Farrell, 2012). It is the first human oncogenic virus discovered and is associated with the development of Burkitt's lymphoma, Hodgkin's lymphoma, nasopharyngeal carcinoma, gastric carcinoma and more (Thompson and Kurzrock, 2004, zur Hausen et al., 1970). Primary EBV infections are usually asymptomatic and mild in children but after adolescence, it commonly causes infectious mononucleosis with symptoms including extreme fatigue, fever, head and body aches, and swollen spleen and lymph nodes. When symptoms are resolved, EBV persists for a lifetime by remaining latent in memory B lymphocytes (Fujiwara et al., 2014). However, EBV can be reactivated by prolonged psychological stress, hormonal changes, infections, and other factors that result in weakened cellular immunity (Murata et al., 2021, Sausen et al., 2021, Dochi et al., 2022, Aiello et al., 2010). This reactivation is associated with autoimmune disease, chronic fatigue syndrome and various other malignancies (Kerr, 2019). Healthy individuals are mostly asymptomatic to EBV reactivation, however immunosuppressed individuals can experience the same symptoms as their primary infection of EBV (Kerr, 2019). Here, we aim to determine if COVID infection can promote EBV reactivation, which could result in complicating symptoms of COVID illness.

**Fig. 1:**
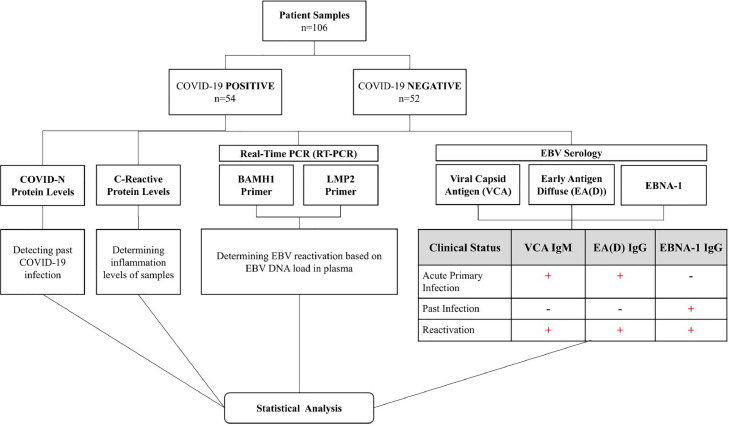
Study Methodology.

After primary EBV infection is resolved and latency is established, antibodies against the latent EBV nuclear antigen-1 (EBNA-1) are produced (Murata et al., 2021, Vouloumanou et al., 2012). Anti-EBNA-1 IgG antibodies are not present during the acute phase of EBV but become detectable 2-4 months after infection and persist for life. Detection of EBNA-1 IgG indicates a past infection. EBV reactivation results in expression of lytic gene products such as the viral capsid antigen (VCA) and early antigen-diffuse (EA-D). Anti-VCA IgM is detectable early in infection and reactivation but falls to undetectable levels in approximately 6 weeks. Anti-EA-D IgG also appears early after infection (3-4 weeks) and reactivation and typically falls to undetectable levels in approximately 4 months (Lennette, 1987). Detection of antibodies against VCA IgM and EA-D IgG is an indicator of EBV reactivation (Gulley, 2001) (Fig. 1). However, diagnosing EBV reactivation on serology alone can produce different results depending on the patient's disease course and the instability of anti-EBV antibodies before the appearance of symptoms (Gold et al., 2021). EBV DNA is frequently detectable in plasma during early infection and reactivation (Lam et al., 2018). qPCR detection of EBV DNA is more sensitive than serology in terms of evaluating reactivation (She et al., 2007).

This study aims to determine if COVID-19 positive patients experience increased levels of EBV reactivation compared to COVID-19 negative patients and differs from previous studies in that samples were collected at a time when the Omicron variant was the dominant strain in the area (New York City Department of Health and Mental Hygiene 2022). Past reports on EBV reactivation were conducted largely before the Omicron variant emerged (Naendrup et al., 2022, Paolucci et al., 2021, Saade et al., 2021, Simonnet et al., 2021, Zubchenko et al., 2022, Rahimi and Talebi Bezmin Abadi, 2022, Im et al., 2022, Brooks et al., 2022, Chen et al., 2021, Meng et al., 2022, Vigon et al., 2021, Xie et al., 2021). Plasma samples from hospital patients determined to be COVID negative or COVID positive using PCR-based testing were studied. EBV DNA load was quantitated and serology toward EBV lytic genes were used as determinants of EBV reactivation (Fig. 1).

## 2 Materials and methods

### 2.1 Sample collection

106 whole blood samples were collected from different individuals treated at Westchester Medical Center, Valhalla, NY, between January 13, 2022, and March 23, 2022, and placed in EDTA tubes. Whole blood samples were spun down to collect plasma. 54 samples were from patients who tested positive for COVID-19 and 52 samples were from patients who tested negative for COVID-19 as determined by PCR-based testing by hospital staff. This IRB exempt study includes samples set to be discarded that were de-identified and marked only as COVID positive or COVID negative and given to the research team. No identifying information or patient data was supplied. Samples were collected during a time frame when the Omicron variant was the most dominant in the NYC area (New York City Department of Health and Mental Hygiene 2022).

### 2.2 Quantification of EBV DNA

DNA from 200 μL of plasma was extracted using the DNeasy Blood & Tissue Kit (Qiagen). TaqMan primer sets were used for EBV quantification targeting the BamH1W and LMP2 regions of the EBV genome and were purchased from IDT (Ryan et al., 2004). TaqMan probes include a 5’ reporter FAM (520 nm emission) and double quencher ZEN/IBFQ.

BamHIW1 Forward primer 5’ GCAGCCGCCCAGTCTCT 3’

BamHIW1 reverse primer 5’ ACAGACAGTGCACAGGAGACT

BamHIW1 TaqMan probe 5’- FAM-AAAAGCTGGCGCCCTTGC 3’ ZEN/IBFQ

LMP2 forward primer 5’ AGCTGTAACTGTGGTTTCCATGAC 3’

LMP2 reverse primer 5’ GCCCCCTGGCGAAGA G 3’

LMP2 TaqMan probe 5’-FAM-CTGCTGCTACTGGCTTTCGTCCTCTGG 3’ ZEN/IBFQ

qPCR conditions were 95°C for 2 min 95°C for 15 sec., 60°C for 1 min. for 45 cycles using QuantStudio™ 5 from Applied Biosystems. Positive and negative controls were included, and a cutoff of CT 40 was used. After cycle 45 negative controls were undetectable, indicating that samples detected at cycle 40 represented levels at least 5 cycles (or 32-fold) beyond background. Detection of anti-ENBA1 IgG antibodies was used as a marker of past infection. To determine percent reactivation, samples which were not positive for EBNA-1 were excluded (10 total samples).

### 2.3 Enzyme-linked immunosorbent assay (ELISA)

#### 2.3.1 EBNA-1 IgG, EA-D IgG, VCA IgM

The presence of antibodies against EBNA-1 IgG, EA-D IgG, and VCA IgM were determined using ELISA kits from Abnova and were performed according to the manufacturer's protocols. To ensure the validity of the assay, positive and negative controls provided in the kit were used. Valid runs had positive controls with an index absorbance range between 2.3-4.2 AU and negative controls below 0.9 AU. The cutoff was calculated by multiplying the mean of calibrators and the calibration factor. Cutoffs were divided from each sample absorbance to get their positivity index. Positive samples have a positivity index >1.10 AU while negatives are <0.90 AU.

### 2.4 C-reactive protein (CRP)

The relative quantification of CRP concentration was determined using a Human C-Reactive Protein ELISA Kit (Sigma-Aldrich). Samples were diluted 200,000-fold and analysis was performed according to manufacturers’ protocol.

### 2.5 COVID nucleoprotein (Np)

Both COVID-19 positive and negative patient samples were quantified for antibodies against COVID Np to detect past SARS-CoV-2 virus infection. The samples were prepared according to the COVID-19 N Human IgG Indirect ELISA Kit (RayBio®) protocol. A cutoff of 6.5 U/ml was established and samples with values below 6.5 were listed as “Undetected” in Table 1 and excluded from analysis in Fig. 3C.

**Table 1:** Test results for COVID negative and COVID positive patients.

Negatives								Positives							
Sample	qPCR BamHI	qPCR LMP2	CRP	EBNA-1	EA(D)	VCA	N Protein	Sample	qPCR BamHI	qPCR LMP2	CRP	EBNA-1	EA(D)	VCA	N Protein
46	Undetected	Undetected	26.6	+	-	-	Undetected	63	Undetected	Undetected	44.8	+	-	-	Undetected
54	Undetected	Undetected	2951.5	+	+	+	Undetected	69	Undetected	Undetected	1466.5	+	+	-	Undetected
59	Undetected	Undetected	1548.4	+	+	-	12.948	78	26.145	29.12241936	4544.4	+\-	+	-	83.575
85	Undetected	Undetected	56.6	+	no sample	+	7.718	82	Undetected	Undetected	3247.5	+	+\-	-	16.628
95	Undetected	Undetected	222.7	+	-	-	7.186	102	Undetected	Undetected	3379.9	+	-	-	Undetected
164	Undetected	Undetected	6.7	+	-	-	Undetected	163	36.923	Undetected	1191.2	+	-	-	20.2
166	Undetected	Undetected	9.7	-	+	-	6.511	194	Undetected	Undetected	154.6	+	-	-	23.539
168	Undetected	Undetected	9.5	+	-	-	Undetected	196	37.197	Undetected	1260.4	+	-	-	45.169
172	36.482	Undetected	8087.6	+	-	-	Undetected	198	36.421	Undetected	594.5	+	-	-	Undetected
179	Undetected	Undetected	510.7	+	+	-	12.451	200	Undetected	Undetected	58.3	+	+	-	22.528
65	Undetected	Undetected	1083.8	+	+\-	-	19.653	75	Undetected	Undetected	1532	+	-	-	6.599
68	Undetected	Undetected	2122.7	+	-	-	no sample	84	Undetected	38.75872803	553.1	+	-	-	372.336
73	Undetected	Undetected	232.9	+	+	-	Undetected	115	Undetected	Undetected	919.2	+	-	-	41.382
76	Undetected	Undetected	26.7	+	+	-	Undetected	116	Undetected	Undetected	645.2	+	-	-	34.637
80	Undetected	Undetected	43.1	+	+	-	Undetected	122	33.331	38.16422653	286.9	+	-	-	30.567
58	Undetected	Undetected	9.5	+	-	-	Undetected	49	Undetected	Undetected	20.1	+	-	-	Undetected
64	Undetected	Undetected	1135	+	-	-	Undetected	50	Undetected	Undetected	468.8	+	-	-	178.322
72	30.186	34.28359222	35.4	+	-	-	Undetected	55	30.478	34.87576675	Undetected	+	no sample	-	Undetected
74	Undetected	Undetected	1024.6	+	+	-	27.284	118	Undetected	Undetected	46.4	+	-	-	56.7
90	35.134	37.69936752	80.8	+	-	-	Undetected	120	35.869	Undetected	1881.4	-	-	-	Undetected
51	Undetected	Undetected	214	+	+/-	-	57.682	114	Undetected	Undetected	3.6	+	-	-	49.225
53	Undetected	Undetected	1491.6	+	-	-	Undetected	117	Undetected	Undetected	Undetected	+	no sample	-	35.822
57	Undetected	Undetected	1728.6	-	-	-	Undetected	123	Undetected	Undetected	15.6	+	-	-	84.071
62	Undetected	Undetected	60	+	-	+	780.647	124	Undetected	Undetected	120.4	+	+	-	35.339
79	Undetected	Undetected	39.2	+	-	-	Undetected	126	38.546	Undetected	43	+	+/-	-	33.74
45	Undetected	Undetected	483.6	+	-	-	Undetected	125	Undetected	Undetected	20.4	+	-	-	279.828
48	Undetected	Undetected	72.8	+	-	-	Undetected	128	Undetected	Undetected	219	+	+	+	47.561
56	Undetected	Undetected	71.8	+	+	-	7.771	133	Undetected	Undetected	587.6	-	-	-	19.985
60	Undetected	Undetected	768.4	+	+/-	-	Undetected	136	35.145	36.49794769	1049.8	+	+	-	54.595
61	Undetected	Undetected	2133	+	-	-	Undetected	139	36.752	Undetected	861.8	+	+	-	62.035
131	31.656	35.08949661	2133	-	-	-	Undetected	129	Undetected	39.97701645	775.2	+	+	-	Undetected
134	Undetected	Undetected	664	+	+/-	-	8.645	138	Undetected	Undetected	479.2	+	+	-	Undetected
165	Undetected	Undetected	6	+	+/-	-	25.352	140	Undetected	Undetected	1710.8	+	-	-	215.617
167	37.5962944	Undetected	1438.8	+	+	-	Undetected	143	29.645	33.68924332	2688.6	+	+	+	28.817
177	Undetected	Undetected	54.2	+	-	-	Undetected	147	Undetected	Undetected	26.4	+	-	-	Undetected
171	Undetected	Undetected	40.6	+	-	-	Undetected	127	Undetected	Undetected	1707.6	+	-	-	7.849
173	Undetected	Undetected	12.8	+	-	-	7.859	142	Undetected	Undetected	281.2	+	+/-	-	8.746
175	Undetected	Undetected	8.8	+	-	-	24.327	152	Undetected	Undetected	19.4	+	-	-	65.977
176	Undetected	Undetected	7.6	+	-	-	18.832	158	Undetected	Undetected	1211.2	+	-	-	Undetected
187	37.096	37.981	358.6	+	+	-	17.92	161	Undetected	Undetected	34.6	+	-	-	Undetected
170	Undetected	Undetected	13.8	+	-	-	36.545	144	Undetected	Undetected	187.4	+	-	-	Undetected
174	Undetected	Undetected	1356.2	+	-	-	Undetected	150	Undetected	Undetected	6.2	+	-	-	Undetected
178	Undetected	Undetected	315	+	-	-	9.457	155	Undetected	Undetected	2087.2	+	+	-	59.019
182	Undetected	Undetected	326.8	+	-	-	25.194	157	Undetected	Undetected	6.2	+	+	-	26.006
189	Undetected	Undetected	1899.4	+	-	-	Undetected	159	35.39	37.545	683.4	+	-	-	148.646
181	37.451	38.086	1963	+	-	-	Undetected	149	Undetected	Undetected	5.4	-	-	-	Undetected
183	Undetected	Undetected	6.4	+	+	-	Undetected	153	Undetected	Undetected	583	+	-	-	29.974
184	Undetected	Undetected	360.6	+	-	-	Undetected	154	Undetected	Undetected	553.8	+	+/-	+\-	77.32
185	Undetected	Undetected	36.4	+	-	-	Undetected	156	36.199	39.246	1485	+	+	-	Undetected
188	Undetected	Undetected	167.6	-	-	-	Undetected	192	Undetected	Undetected	23.6	-	+/-	-	Undetected
190	Undetected	Undetected	552.2	+	-	-	16.409	193	Undetected	Undetected	83.6	+	-	-	340.319
191	Undetected	Undetected	13.6	+	-	-	Undetected	195	Undetected	Undetected	29.2	-	-	-	17.152
								201	Undetected	Undetected	10.4	+	-	-	Undetected
								202	Undetected	Undetected	70	+	-	-	29.742

### 2.6 Statistical analysis

**Fig. 2:**
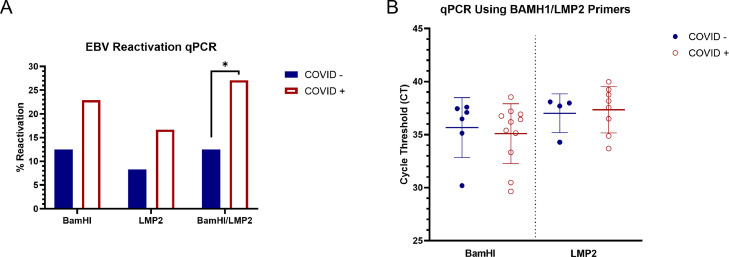
Increased EBV reactivation in COVID-19 patients. The presence of viral genomes in patient serum was determined using BamHI and LMP2 primers to target EBV DNA. A) Samples testing positive for the presence of EBV DNA in serum are chartered as percentage of samples where EBV was reactivated among COVID negative and positive groups using BamHI, LMP2 or both primer sets. Two sample proportion Z test: BamHI: p=0.0906, LMP2: p=0.1085, BamHI/LMP2: p=0.0364. * represents p<0.05. B) Relative quantitation of EBV genome copies using BamHI and LMP2 primers sets. Welch's t-test with BamHI primers (p=0.8223). Welch's t-test with LMP2 primers (p=0.9436).

**Fig. 3:**
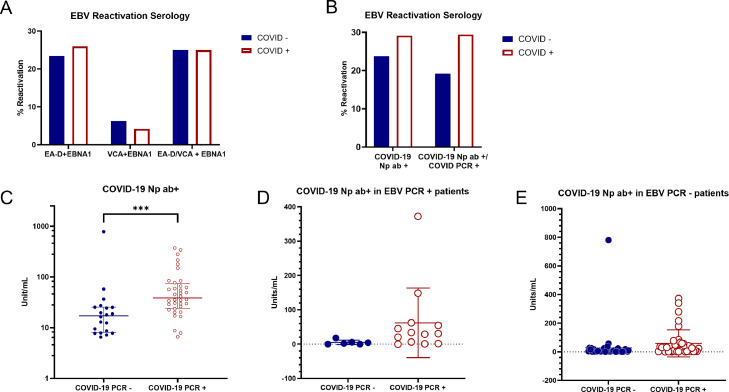
Detection of EA-D IgG and VCA IgM as determinants of EBV reactivation. A) Samples testing positive for the presence of EBV EA-D IgG and VCA IgM are charted as percentage of samples where EBV was reactivated among COVID negative and positive groups determined by PCR assay. Two sample proportion Z test: EBNA-1 and EA-D: p=0.3821, EBNA-1 and VCA: p=0.6593, EBNA-1 and EA-D/VCA: p=0.4519. B) Samples testing positive for the presence of either EBV EA-D IgG and VCA IgM, in addition to EBNA-1 IgG, are chartered as percentage of samples where EBV was reactivated among COVID negative and positive groups where COVID status was determined by the detection of COVID anti-nucleoprotein (COVID Np ab+) (left side) or detected by either PCR or detection of antibodies against nucleoprotein (right side). Two sample proportion Z test: COVID-19 positivity determined by Np seropositivity: p=0.2815, COVID-19 positivity determined by a positive PCR test or Np seropositivity: p=0.1588. C) Relative levels of antibodies against Np among COVID negative and COVID positive patients. COVID +/- groups on X-axis represent COVID determination by PCR test; therefore, COVID negative patients with detection of antibodies against Np demonstrate past infection. COVID status determined by PCR: p=0.0002 (Mann-Whitney test). *** represents p<0.001. D) Anti-Np IgG levels in EBV reactivated patients. Samples with antibodies against COVID Np were graphed for patients found to have reactivated EBV determined by detection of EBV genomes. EBV reactivation patients with current COVID determined by PCR had higher levels of antibodies against Np than EBV reactivating patients who had a past infection (p=0.066 Welch's t-test). E) Anti-Np IgG levels in EBV PCR negative patients. COVID PCR+ patients had higher levels of antibody against Np than COVID PCR – patients in the absence of EBV reactivation (EBV PCR-) (p=0.233 Welch's t-test).

**Fig. 4:**
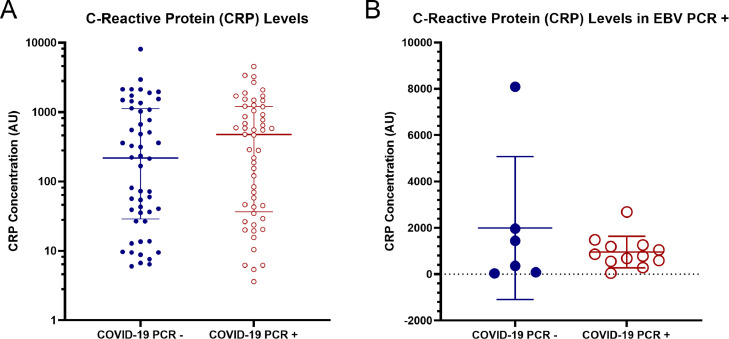
A) Measurement of CRP levels among COVID positive and negative groups: (p=0.4691 Mann-Whitney test). B) Measurement of CRP levels among COVID positive and negative groups determined via PCR among patients with EBV reactivation determined by EBV genome detection: (p=0.4508 Welch's t-test).

Welch's t-test (Figs. 2B, 3D, 3E, 4B), Mann-Whitney test (Figs. 3C and 4A) and two sample proportion Z test (Figs. 2A, 3A, 3B) were used to calculate significant difference. GraphPad Prism 9 was used to construct graphs and conduct statistical analysis. Summary of raw data is shown in Table 1.

## 3 Results

### 3.1 Quantitative PCR detection of EBV

SARS-CoV-2 infection results in increased EBV reactivation. Detection of COVID-19 resulted in increased EBV reactivation as determined by detection of EBV DNA in plasma. To distinguish primary infection from reactivation, past infection status must first be determined. EBNA-1 IgG is an indicator of previous EBV infection. Samples that did not test positive for the presence of EBNA-1 IgG were excluded from analysis and are likely primary infections, not a result of reactivation. 19/96 samples (19.8%) showed reactivated EBV based on detection of EBV genomes with TaqMan probes and at least one primer set (BamHI and LMP2). 13/48 (27.1%) of reactivations were from the COVID positive group, while only 6/48 (12.5%) of reactivations belonged to the negative group. 17/96 (17.7%) were detected using BamHI primers with 6/48 (12.5%) reactivated in the COVID negative group, and 11/48 (22.9%) reactivated in the COVID positive group. qPCR using the LMP2 primer set showed the reactivation of 12/96 samples (12.5%) with 4/48 (8.3%) reactivated in the COVID negative group, and 8/48 (16.7%) reactivated in the COVID positive group (Fig. 2A). 10/12 samples found to be reactivated with LMP2 were also detected with BamHI primers demonstrating strong overlap. For detection of EBV genomes in the plasma samples, 45 PCR cycles were performed to detect EBV DNA using TaqMan probes. In order to distinguish true positives from background levels, a cut-off of cycle 40 was used for inclusion as EBV reactivation determined by qPCR DNA detection. This represents a minimum 32-fold increase above background levels, drastically reducing the possibility of false positives. Negative controls were undetectable at cycle 45. Taken together the data shows that the incidence of EBV reactivation is increased in COVID patients.

No statistical difference was found in the amount of EBV genomes detected in the plasma of reactivated COVID negative and COVID positive patients (Fig. 2B). The mean CT value of the COVID negative group (35.66 CT) was not statistically significant (p=0.8223) compared to the COVID positive group (35.09 CT) with BamHI primers. The mean CT values of EBV reactivated samples detected with LMP2 primers were very similar between the COVID negative group (37.01 CT) and positive (37.34 CT) group (p=0.9436). This data indicates that, in this study, the number of EBV genomes produced via reactivation do not differ significantly between COVID positive and negative patients.

### 3.2 EBV serology

96/106 patients (90.56%) had detectable levels of anti-EBNA-1 IgG, indicative of a past EBV infection. 25/103 (24.3%) were positive for anti-EA-D IgG and only 5/106 (4.71%) had anti-VCA IgM antibodies. 23/93 (24.7%) samples were positive for both EBNA-1 and EA-D, 12/46 (26.0%) of which were COVID positive and 11/47 (23.4%) were negative. 3/48 (6.3%) were positive for EBNA-1 and VCA from the COVID negative group and 2/48 (4.2%) were positive from the COVID positive group. When either EA-D or VCA was detected in combination with EBNA-1, 12/48 (25.0%) COVID negative patients showed reactivation compared to 12/48 (25.0%) in the COVID positive group (Fig. 3A). There was no significant difference in EBV reactivation as determined by serology against VCA and EA-D antibodies when the COVID negative and positive groups were defined by PCR assay.

To determine if a patient had a past SARS-CoV-2 infection, we quantified antibodies against the COVID nucleoprotein. Most COVID-19 patients develop IgG antibodies within 2–3 weeks after symptom present (Van Elslande et al., 2021). COVID-19 anti-Np IgG antibodies can be detected as early as 7 -10 days after infection and remain for at least several months, whereas PCR-based testing for COVID-19 DNA may be detected 0-4 days after symptoms begin and may remain for several weeks (Koc et al., 2022, Centers for Disease Control and Prevention 2022, Mallett et al., 2020). 20/52 of the patient samples that were determined to be COVID negative based on PCR were found to have antibodies against COVID Np, indicating they had a past infection. 36/54 COVID positive samples (as determined by PCR) were positive for antibodies against Np. Overall, 56/106 (52.8%) patients had a previous or current SARS-CoV-2 infection as determined by antibody against Np.

When seropositivity for anti-Np is used as the indicator for COVID-19 detection, there is an increasing trend in EBV reactivation determined through detection of antibodies against EA-D and VCA in COVID-19 positivity samples. 9/38 (23.7%) patient samples were negative for anti-Np antibodies but had antibodies against either EA-D or VCA, whereas 16/55 (29.1%) patient samples were positive for anti-Np antibodies and had antibodies against either VCA or EA-D. When COVID-19 positivity is determined via positive PCR test or Np seropositivity EBV reactivation is also observed more in COVID-19 positive samples (5/26 (19.2%) vs 20/68 (29.4%)) (see Fig. 3B).

COVID positive samples had a median of 38.60 unit/mL of anti-Np antibodies, while negatives had a median of 17.16 unit/mL as determined by ELISA (Fig. 3C). The positive and negative groups have a statistically significant difference according to the Mann-Whitney test (p=0.0002), indicating an increase in Np antibodies for COVID PCR+ positive patients.

The relationship between anti-Np IgG levels in EBV reactivated patients was evaluated. Samples with antibodies against COVID Np protein for patients found to have reactivated EBV are shown in Fig. 3D. Reactivated EBV was determined by detection of EBV DNA via either BamHI or LMP2 primers. COVID +/- groups represent COVID determination by PCR test. COVID negative patients with detection of antibodies against Np indicates a past infection. Interestingly, EBV reactivation patients with current COVID, determined by PCR, had higher levels of antibodies against Np than EBV reactivating patients who had a past infection (5.0 vs 61.9 average mean (p=0.066)) (Fig. 3D).

Similarly, the relationship between anti-Np IgG levels in patients that did not experience EBV reactivation was also investigated among the COVID negative and positive groups (Fig. 3E). COVID PCR + patients had higher levels of antibodies against Np than those without current COVID (28.4 vs 58.5 average mean (p=0.223)). While Fig. 3D and E both show increased detection of antibodies against Np in individuals with PCR+ COVID, the difference in Np antibody levels among COVID positive and COVID negative group is much larger in the EBV reactivated patients. However this difference may be due to one sample with a very high level of antibodies against Np.

### 3.3 C-reactive protein

COVID-19 positive and negative patient samples were monitored for CRP levels. The COVID positive samples have a higher median of 474 AU compared to the negative group's median of 218.4 AU. The difference of medians in the two groups is not statistically significant according to the Mann-Whitney test (p=0.4691) (Fig. 4A). There was no correlation between CRP levels and COVID positive vs. negative patients. Additionally, no correlation between CRP levels and EBV reactivation between COVID -/+ groups was found (Fig. 4B). COVID negative samples had a mean of 1994 AU and the COVID positive group's mean is 956 AU (p=0.4508).

## 4 Discussion

Several studies have investigated the relationship between EBV and COVID. A study conducted in Wuhan, China observed that 55.2% of hospitalized COVID-19 patients were positive for EBV reactivation based on the presence of antibodies against VCA IgM (Chen et al., 2021). A recent observational case-control study conducted in Italy saw 95.2% of COVID-19 ICU patients and 83.6% SICU patients were positive for EBV reactivation. Their comparison between the two groups suggested a correlation between EBV DNA load and COVID severity (Paolucci et al., 2021). Saade et al found 56.1% of EBV reactivations in severe COVID-19 patients after admission to the ICU (Saade et al., 2021) and another reported increased antibodies against EBV and detectable viremia in plasma in critical COVID patients (Vigon et al., 2021).

EBV reactivation has also been examined in the context of long COVID. As of 2022, the CDC determined that 1 in 5 adults in the U.S. who were previously infected with COVID-19 experienced long COVID conditions (Centers for Disease Control and Prevention 2022). Long COVID claimed over 3500 lives in America between January 2020 and June 2022 (Centers for Disease Control and Prevention 2022). The most persistent symptoms in patients hospitalized due to long COVID are fatigue, dyspnoea, loss of memory, and sleep disorders (Garrigues et al., 2020). A 2021 retrospective study observed 66.7% of long COVID patients were positive for EBV based on the presence of EA-D and VCA IgM antibodies (Gold et al., 2021). Another study by Zubchenko et al found EBV reactivation, determined by PCR detection of EBV DNA in peripheral blood, in 42.6% of long COVID patients (Zubchenko et al., 2022). Peluso et al reported that EBV reactivation is associated with higher odds of long COVID symptoms (Peluso et al., 2023).

These previous studies focused on patient populations that were infected with a variants prior to Omicron. Our samples were collected when the Omicron variant was the most dominant in NYC cases (New York City Department of Health and Mental Hygiene 2022) which may result in differences from past studies. To our knowledge this is the first report investigating EBV reactivation due to COVID during the Omicron surge. This study analyzed EBV in samples from hospital patients that are positive and negative for COVID-19 to determine whether EBV reactivation is triggered by COVID-19 irrespective of disease severity, whereas many previous studies focused on severe or long COVID. Patient samples from this study likely represent the full spectrum of COVID infection, ranging from asymptomatic, to mild cases, to severe cases and long COVID. Herein we use both serology and EBV genome detection to analyze EBV reactivation and include detection of EBNA-1 to differentiate primary EBV infection from reactivation. Additionally past SARS-Cov-2 infections were detected by detecting antibodies against the SARS-CoV-2 Np.

Using two primer sets for determining EBV reactivation, it was found that the COVID positive group resulted in significantly increased reactivation of EBV (27.1% vs 12.5%) compared to the negative group. It should be noted that our COVID negative group does not represent a healthy population and rather that of patients treated for various unknown reasons at Westchester Medical Center. Interestingly, the 12.5% reactivation among non-COVID patients found in this study is similar to the amount reported in a 2016 study among a cohort of patients (12%) treated at Johns Hopkins Hospital with no current, prior, or subsequent EBV disease (Kanakry et al., 2016). While we did not have access to a healthy population for this study, other groups have reported EBV reactivation rates of 0.6% and 3% among healthy and immunocompetent individuals as determined by PCR of serum samples (Kanakry et al., 2016, Walton et al., 2014).

The mean CT for EBV genome copy detection of the COVID positive (35.09 CT) and negative group (35.66 CT) was not statistically significant (p=0.8223) using primers targeting BamHI. Paolucci et al reported increased EBV genome copy number in severe COVID cases (patients in ICU) compared those in less severe cases (sub-ICU) (Paolucci et al., 2021). While we did not observe an increase in EBV DNA in the serum, we did detect approximately a 2-fold increase in the number of people with EBV reactivation among COVID positive patients.

The serology of EBNA-1 antibody presence in samples is in line with what can be seen in the general population (approximately 90%) (Tzellos and Farrell, 2012). The serology for EA-D and VCA antibodies did not differ between the COVID positive and negative groups when COVID status is determined by PCR. However, when COVID status was determined by detection of Np there was a noticeable increase in EBV reactivation as determined by serology for VCA and EA-D. Differences accounting for EBV reactivation determined by PCR vs serology are likely due to the time course of disease and initial production and duration of antibodies produced. The presence of EBV DNA-containing particles is likely cleared by the immune system before EA-D and VCA antibodies are produced. EBV reactivation results in the presence of EBV particles in the extracellular serum, therefore detection of EBV DNA via qPCR is likely a better indicator of reactivation.

CRP is a biochemical marker of inflammation (Pepys and Hirschfield, 2003). An increase in its concentration is also associated with the severity of COVID-19 (Ali, 2020). A 2021 study found higher CRP levels in patients with both EBV and COVID-19 compared to patients with only COVID-19 (Chen et al., 2021). However, another study conducted when the alpha strain was dominant, found no association between EBV reactivation and elevated CRP (Brooks et al., 2022). When comparing data from this study we also found there was no statistical difference in CRP levels among COVID positive patients with and without EBV reactivation. COVID positive EBV reactivated patients had a mean CRP level of 956 AU and the COVID positive population without EBV reactivation had a CRP mean of 712 AU (p=0.357). Perhaps these variations are due to differences in COVID variants and disease severity of patients included in the studies. In this study, according to the comparison between COVID positive and negative groups, there is also not a significantly higher (p=0.4691) CRP level for patients with COVID-19. Since we do not have a healthy population, this could be due to other CRP-elevating conditions.

High levels of antibodies against COVID Np can indicate patients that have been infected within weeks to 3 to 6 months prior. 20/52 of the COVID negative patients (38.5%) were identified to have been previously infected with SARS-CoV-2. Therefore, if these patients experience EBV reactivation, it is reasonable to consider COVID-19 as one of the possible causes. In this study it was found that EBV reactivation patients with current COVID-19 had higher levels of antibodies against Np than EBV reactivating patients who had a past infection (Fig. 3D). A study by Imai et al found that more severe COVID correlates with elevated anti-Np antibodies (Imai et al., 2021) and this data taken together could suggest that COVID severity correlates with increased EBV reactivation. The COVID negative group's significantly lower (p=0.0002) antibodies against COVID Np is also in line with the fact that these levels gradually decrease after the infection has been resolved.

Our results point to a trend suggesting that COVID-19 reactivates EBV at a higher rate than non-COVID patients. Significance of this work is heightened by studies of hospitalized COVID patients showing that reactivated EBV significantly increased mortality when compared to EBV negative patients (Xie et al., 2021, Manoharan and Ying, 2023). In addition, it was found that patients with more severe pneumonia had EBV viremia (Im et al., 2022). Results of this work may help determine the course of treatment for COVID positive patients experiencing EBV reactivation. To this end, Meng et al found that patients experiencing EBV reactivation due to COVID showed increased survival outcomes when treated with the EBV inhibitor, ganciclovir (Meng et al., 2022).

## Funding

This work was supported by a startup grant from 10.13039/100016250New York Medical College.

## CRediT authorship contribution statement

**Keishanne Danielle E. Bernal:** Conceptualization, Methodology, Investigation, Formal analysis, Writing – review & editing. **Christopher B. Whitehurst:** Conceptualization, Methodology, Investigation, Formal analysis, Writing – review & editing, Supervision.

## Declaration of Competing Interest

The authors declare that they have no known competing financial interests or personal relationships that could have appeared to influence the work reported in this paper.

## Data Availability

Data will be made available on request.
